# Examining the intersection of cognitive and physical function measures: Results from the brain networks and mobility (B-NET) study

**DOI:** 10.3389/fnagi.2023.1090641

**Published:** 2023-02-02

**Authors:** Atalie C. Thompson, Michael E. Miller, Elizabeth P. Handing, Haiying Chen, Christina E. Hugenschmidt, Paul J. Laurienti, Stephen B. Kritchevsky

**Affiliations:** ^1^Department of Surgical Ophthalmology, Wake Forest School of Medicine, Winston-Salem, NC, United States; ^2^Section on Gerontology and Geriatric Medicine, Department of Internal Medicine, Sticht Center for Healthy Aging and Alzheimer’s Prevention, Wake Forest School of Medicine, Winston-Salem, NC, United States; ^3^Division of Public Health Sciences, Wake Forest School of Medicine, Winston-Salem, NC, United States; ^4^Department of Human Development and Family Studies, Colorado State University, Fort Collins, CO, United States; ^5^Department of Biostatistics and Data Sciences, Wake Forest School of Medicine, Winston-Salem, NC, United States; ^6^Department of Radiology, Wake Forest School of Medicine, Winston-Salem, NC, United States

**Keywords:** cognitive function, mobility, aging, canonical correlation analysis, physical function

## Abstract

**Background and objectives:**

Although evidence exists that measures of mobility and cognition are correlated, it is not known to what extent they overlap, especially across various domains. This study aimed to investigate the intersection of 18 different objective cognitive and physical function measures from a sample of unimpaired adults aged 70 years and older.

**Research design and methods:**

Canonical correlation analysis was utilized to explore the joint cross-sectional relationship between 13 cognitive and 6 physical function measures in the baseline visit of the Brain Networks and Mobility Function (B-NET) Study (*n* = 192).

**Results:**

Mean age of participants was 76.4 years. Two synthetic functions were identified. Function 1 explained 26.3% of the shared variability between the cognition and physical function variables, whereas Function 2 explained 19.5%. Function 1 termed “cognitive and physical speed” related the expanded Short Physical Performance Battery (eSPPB), 400-m walk speed, and Dual Task gait speed measures of physical function to semantic fluency animals scores, Digit Symbol Coding (DSC), and Trail Making Test B. Function 2 termed “complex motor tasks and cognitive tasks” related the Force Plate Postural Sway Foam Task and Dual Task to the following cognitive variables: MoCA Adjusted Score, Verbal Fluency L words, Craft story immediate and delayed recall, and Trail Making Test B.

**Discussion and implications:**

We identified groups of cognitive and physical functional abilities that were linked in cross-sectional analyses, which may suggest shared underlying neural network pathway(s) related to speed (Function 1) or complexity (Function 2).

**Translational significance:**

Whether such neural processes decline before measurable functional losses or may be important targets for future interventions that aim to prevent disability also remains to be determined.

## Introduction

Walking is a complex task integrating neuromuscular and cognitive components (Wilson et al., 2019). Several studies have shown that gait speed is associated with cognitive function as measured by a variety of global and domain specific assessments, but the strength and direction of associations have varied depending on the test and cohort, with some studies suggesting slow gait predicts cognitive changes and others that cognitive performance predicts decline in gait speed (Fitzpatrick et al., 2007; Watson et al., 2010; Mielke et al., 2013; Verghese et al., 2013; Morris et al., 2016; Peel et al., 2019; Handing et al., 2021; Jayakody et al., 2021). Many studies include persons with mild cognitive impairment or clinical neurological disease and so some associations may reflect concomitant impairments in both areas. Thus, examining these relationships in a cognitively intact cohort could help to determine whether the observed associations are intrinsic.

Moreover, the majority of the literature examining cognition and physical function to date have focused on gait speed but not particular aspects of physical function that contribute to gait, such as balance, muscle strength, and power. Given the limited range of physical assessments in prior work, it is not clear whether the association of gait and cognition stems from particular components of gait or if these components may differentially relate to different aspects of cognitive function especially on different tests (Clouston et al., 2013; Mignardot et al., 2014; Szturm et al., 2015; Rosano et al., 2016; Bahureksa et al., 2017; Bohannon, 2019; Chou et al., 2019; Wiśniowska-Szurlej et al., 2019; Meunier et al., 2021). A better understanding of the basis of the relationship of cognitive and physical function is needed.

In this analysis, we used canonical correlation analysis (CCA) to describe the patterns of association between 18 different objective measures of physical and cognitive function collected at the baseline visit of the Brain Networks and Mobility Function (B-NET) study. B-NET is a longitudinal study of 192 older adults free of mild cognitive impairment (MCI), dementia, or a clinical history of neurologic disease in order to understand the relationship between functional brain networks involving the sensorimotor cortex and lower extremity mobility function. CCA estimated linear combinations of the cognitive and physical measures in order to maximize the amount of explained shared variance (Sherry and Henson, 2005; Zhuang et al., 2020). Such linear functions describe the intersection of specific cognitive and physical measures, which may reflect shared underlying neural networks that could be considered novel therapeutic targets in future work. While this analysis was exploratory in nature, we expected to replicate prior observations of the association between executive function and gait speed (Fitzpatrick et al., 2007; Watson et al., 2010) as well as better elucidate which particular cognitive functions may be related to specific aspects of physical function.

## Materials and methods

### Study design

This study includes participants in the baseline visit of the B-NET study, an ongoing longitudinal, observational study of community-dwelling older adults aged 70 and older recruited from Forsyth County, NC and surrounding regions (NCT03430427). Participants were excluded from the study if they were a single or double lower extremity amputee, had musculoskeletal impairments severe enough to impede functional testing (e.g., joint replacements), or dependency on a walker or another person to ambulate. The participants were also excluded if they had a history of any of the following: surgery or hospitalization within the past 6 months, serious or uncontrolled chronic disease (e.g., stage 3 or 4 cancer, stage 3 or 4 heart failure, liver failure or cirrhosis of the liver, uncontrolled angina, respiratory disease requiring the use of oxygen, renal failure requiring dialysis, diagnosis of schizophrenia, bipolar, or other psychotic disorders, or alcoholism (>21 drink per week)), clinical manifestation of a neurologic disease affecting mobility, prior traumatic brain injury with residual deficits, brain tumors, seizures within the last year, and major uncorrected hearing or vision problems. In addition, they were excluded if they reported plans to relocate within the next 2 years, were participating in a behavioral intervention trial, or had evidence of impaired cognitive function. Cognitive impairment was defined based on scores on the Montreal Cognitive Assessment (MoCA). MoCA scores of 20 or lower on the MoCA were considered ineligible. The full complement of cognitive tests in those with scores between 21 and 25 was reviewed by the study neuropsychologist, and those with a pattern consistent with MCI were excluded. Each participant signed a written informed consent form and the Institutional Review Board (IRB) of the Wake Forest School of Medicine approved the study.

### Cognitive function testing

#### MoCA

The MoCA is a brief cognitive screening tool for global cognition and is scored out of a possible total score of 30 points, with higher scores indicating better cognitive performance. It assesses different cognitive domains including attention and concentration, memory, language, conceptual thinking, calculations, and orientation (Nasreddine et al., 2005; Freitas et al., 2013). The overall MoCA score was evaluated in this study.

#### Semantic fluency

Semantic fluency is a measure of speeded word retrieval and executive function. The participant is asked to name various items of a given semantic category (animals or vegetables), and the number of unique responses named is scored. Participants are given 60 s to generate as many distinct responses as they can, with a higher score indicating better performance. The individual score for animals or vegetables was evaluated in this study.

#### Verbal fluency

Verbal Fluency is a measure of speeded word retrieval and executive function. The participant is asked to name items that begin with a certain letter of the alphabet (F or L). The number of unique responses named is scored. Participants are given 60 s to generate as many distinct responses as they can, with a higher score indicating better performance. The individual score for F or L was evaluated.

#### Craft story

The Craft Story 21 Recall (Immediate) assesses the ability to recall a short story. The study staff reads a short story and immediately after hearing the story, the participant is asked to retell the story from memory. Points are given for correct recall of details from the story. After approximately a 20-min delay, the participant is asked to repeat the story and scored for correct recall of details from the story, with higher scores indicating better performance. The immediate and delayed scores were each assessed in this study.

#### Digit symbol coding

The Digit symbol coding (DSC) assesses processing speed. The participant is asked to translate numbers (1–9) to symbols using a key provided at the top of the test form. The outcome included here is the total number of correct responses within 90 s, with higher scores indicating better performance.

#### Auditory verbal learning test

The Auditory verbal learning test (AVLT) is a 15-word, six trial list learning task with immediate and delayed recall conditions. Fifteen words are read aloud and then the participant must recall the words from the list. Correct words recalled after each trial are awarded 1 point. After a 20- to 30-min delay, the participant is asked to recall the same words from the list again (Schmidt, 1996). The Delayed Recall score is the mean number of words correctly recalled across all six trials, and the Short Delay Recall (Trial 6) reflects the raw number of words recalled after an interference trial, with higher scores indicating better performance. The short and delayed recall scores were considered in this analysis.

#### Trail making A and B

The Trail making (TMT) includes Parts A and B. Part A requires participants to connect a series of circles numbered 1 to 25, and it assesses visual scanning, sequencing, and psychomotor speed. Part B adds a set shifting element by requiring the participant to switch between numbers and letters. The maximum time in seconds is 150 for Part A and 300 for Part B, with higher number of seconds indicating worse performance. TMT A and TMT B scores were each analyzed in this study.

#### Flanker

A computerized assessment of executive function and response inhibition administered using the EPRIME software 2.0 (Psychology Software Tools, Inc.). The Flanker task required participants to indicate the direction, by button press, of a central target arrow flanked by congruent or incongruent arrows. Accuracy and response times were recorded with the differenced in response time between the congruent and incongruent conditions being the summary score (Sanders et al., 2018). Higher difference scores are considered poorer performance. For this analysis, the log of the ratio of median response times was used.

### Mobility function testing

#### Grip strength

Grip strength (kg) was measured using a Jaymar handheld dynamometer. Three trials were performed and the maximum was taken across the 3 trials of the dominant hand, with larger values representing better performance.

#### Postural sway

Postural sway was assessed using Center-of-Pressure (COP) trajectory data collected at 100 Hz using an Advanced Mechanical Technology Incorporated (AMTI) AccuSway biomechanics force platform. Participants were barefoot in an upright closed stance and asked to stand comfortably on the platform for a series of five, 30-s trials. Postural sway was measured using a standard firm force plate as well as a foam force plate. For both plates, the area (in.^2^) within the 95% confidence ellipse path around the center of pressure was used to represent performance, with higher values representing worse performance.

#### Expanded short physical performance battery

The expanded Short Physical Performance Battery (eSPPB) was adapted from the test described by Guralnik et al. (1994) in order to address ceiling effects that could limit the value of the traditional SPPB in a well-functioning cohort such as BNET. The eSPPB increases the challenge to participants’ physical function assessments for balance and gait. Participants are asked to hold a side-by-side posture for 10 s, and the semi-tandem, tandem, and one-leg position for 30 s each. If participants are unable to hold the semi-tandem stand for 30 s, then they are requested to hold a short tandem stand for 10 s instead of 30 s. In addition to the usual 4-m gait speed (m/s), a narrow walking pace is also assessed over 4 m wherein participants are required to keep their steps in between 2 parallel lines marked 20 cm apart. The number of times a participant can stand up from a seated position, or chair pace, is also measured during a 5 s period. Scores for each subcomponent are then calculated based on the proportion of the best possible score (a continuous measure), not according to ranges of performance (a categorical measure). The resulting overall eSPPB score ranges from 0–4, rather than the traditional 12-point right-skewed categorical score distribution of the SPPB. The higher values represent better performance.

#### 400-m walk test

Participants completed the fast-paced 400-m walk protocol developed by the Health Aging and Body Composition study, which has been shown to predict future mobility disability and mortality (Newman et al., 2006). The 400-m gait speed in m/s was analyzed in this study.

#### Dual task

During the Dual Task, participants completed 4 trials of walking over the 4-m GaitRITE Mat while saying the alphabet but skipping every other letter (e.g., B D F H J, etc.). The gait velocity was measured in cm/s and converted to m/s for the purpose of analysis.

#### Statistical methods

Means (SD) and proportions were calculated for descriptive statistics and Spearman correlations were calculated between all cognitive and physical function variables. The distributions of variables were examined, and log transformation was performed for postural sway, 400-m walk pace, Trails A and B. For the main analysis, a canonical correlation analysis (Mielke et al., 2013) (CCA) was used to relate the 12 cognition measures to the 6 physical function measures (see Table 1 for a listing of cognition and physical function variables) after adjusting for sex and years of education (i.e., the CCA was run on the residuals for each variable after removing the sex and education effect). Sex was included as an adjustment factor due to significant associations of sex with strength, especially grip strength, and education was adjusted for since it can affect cognitive performance. This CCA analysis creates linear functions of the two groups of variables that maximize the correlation between the synthetic variables (e.g., one for cognition and one for physical function) formed by those linear functions, with the number of pairs of synthetic variables being equal to the lower number of variables within a group (6 physical function measures in our case). Each synthetic variable is mathematically constructed so that it is uncorrelated with the other synthetic variables, and the canonical correlation *R*_c_ is the Pearson correlation between these linear functions. The square of this value represents the proportion of variance shared between the cognition and physical function variables, after accounting for all previous pairs of synthetic variables. This technique accounts for the correlation structures among the cognitive and physical variables to help elucidate shared aspects of physical and cognitive measures. An advantage of this technique is that it provides this information in a single analysis, reducing concerns over multiple testing that one might have if comparing sets of cognitive and physical measures in a pairwise fashion.

**Table 1:** Descriptive statistics from participants at baseline in the BNET study.

	Overall (*N* = 192)
	Mean (SD); range
Age	76.43 (4.72); 70 to 90
Sex	
Women	108 (56.2)
Men	84 (43.8)
Race/Ethnicity	
Caucasian or White/Non-Hispanic	171 (89.1)
African American or Black/Non-Hispanic	18 (9.4)
Caucasian or White/Hispanic	2 (1.0)
Asian/Non-Hispanic	1 (0.5)
BMI	28.39 (5.63); 15.7 to 59.8
Years of education	15.68 (2.45); 12 to 25
Cognitive measures	
MoCA adjusted score	25.64 (2.20); 21 to 30
Semantic fluency: Animals (no. in 60 s)	18.78 (4.82); 7 to 34
Semantic fluency: Vegetables (no. in 60 s)	13.26 (3.87); 0 to 26
Verbal Fluency: F words (no. in 60 s)	12.33 (3.94); 3 to 26
Verbal Fluency: L words (no. in 60 s)	13.23 (4.02); 4 to 28
Craft immediate recall (no.)	21.03 (5.99); 7 to 35
Craft delayed recall (no.)	18.67 (5.74); 7 to 34
DSC (no. in 90 s)	55.18 (12.20); 21 to 87
AVLT short delay recall, Trial 6 (no.)	8.37 (3.20); 0 to 15
AVLT delayed recall (no.)	7.94 (3.46); 0 to 15
TMT A (sec)	36.75 (11.15); 18 to 89
TMT B (sec) (*N* = 191)	98.70 (43.96); 36 to 300
Flanker (log of ratio of medians) (*N* = 189)	0.11 (0.08); −0.03 to 0.39
Physical function measures	
Maximum grip strength (kg) (*N* = 189)	28.80 (9.78); 8 to 52
Force plate postural sway 95% area (in.^2^) - Firm (*N* = 188)	0.37 (0.34); 0.07 to 2.40
Force plate postural sway 95% area (in.^2^) – Foam (*N* = 188)	1.18 (0.82); 0.34 to 8.68
eSPPB score (*N* = 190)	2.00 (0.52); 0.48 to 3.26
400-m walk pace (m/s)	1.27 (0.43); 0.31 to 4.17
Dual Task pace (m/s) (*N* = 186)	1.07 (0.21);0.55 to 1.67

MoCA, Montreal Cognitive Assessment; DSC, Digit Symbol Coding; AVLT, Auditory Verbal Learning Test; TMT, Trail Making Test.

As suggested by Sherry and Henson (2005) we used both the magnitude of the standardized canonical function coefficients and the structure coefficients (*r*_s_) to inform interpretation of the synthetic variables. Structure coefficients measure the bivariate correlation between an observed individual variable and the synthetic variable that incorporates that measure. The square of the structure coefficients (*r*_s_^2^) measures the proportion of variance shared by the observed variable and the created synthetic variable. The communality coefficient (*h*^2^) measures the proportion of variance that each observed measure shares with the solution across the selected functions, is equal to the sum of the structure coefficients, and is informative as to the importance of the individual variable across the selected functions. The Wilks lambda criterion was used to test the full model and perform hierarchal tests of groups of functions. We followed the recommendations of Sherry and Henson in our presentation of CCA results.

## Results

Descriptive statistics for demographic characteristics, cognitive measures, and physical function measures are presented in Table 1. To explore the multivariate shared relationship between cognition and physical function, the CCA was conducted using data from 174 participants with complete data for the 12 cognition variables and the 6 physical function variables. Twenty participants did not have complete data and therefore were excluded from the main analyses. Six functions were obtained with squared canonical correlations of 0.263, 0.195, 0.133, 0.098, 0.053, 0.012 for each, respectively. We found that the full model incorporating all six functions was statistically significant with Wilks’s *λ* = 0.434, *F* (78, 838.7) = 1.76, and *p* = 0.0001. Moreover, the full model explained 57% (1-λ × 100) of the shared variance between the variable sets. In contrast, hierarchal tests for functions 2–6 had *p* = 0.021, functions 3–6 had *p* = 0.25, and all remaining hierarchal groups had *p* ≥ 0.62. Function 1 explained 26.3% of the shared variability between the cognition and physical function variables, whereas Function 2 explained 19.5% of the remaining variance in the variable sets after accounting for the variability explained by the first function. All remaining functions each explained ~13% or less of the remaining variance. We focused our results presentation on the first two functions.

Standardized canonical function coefficients, structure coefficients (*r*_s_), squared structure coefficients (*r*_s_^2^) and communalities (*h*^2^) are presented in Table 2 for Functions 1 and 2. Function 1 was labeled as a “cognitive and physical speed” variable because the primary cognitive and physical measures in this canonical correlation reflected speed of performance. Inspection of the coefficients for Function 1 revealed that the important physical function variables were primarily eSPPB score, log transformed 400-m walk speed, and the Dual Task pace. These variables had the largest squared structure coefficients (all ≥43.2). Note that the sex- and education-adjusted, Pearson correlation coefficients between the eSPPB and the log transformed 400-m walk speed (*r* = 0.58), and Dual Task (*r* = 0.62) were fairly large (Supplementary Table S1).

**Table 2:** Canonical correlation analysis results adjusted for sex (*n* = 174).

	Function 1			Function 2			*h*^2^ (%)
	Standardized coefficient	*r_s_*	*r_s_^2^* (%)	Standardized coefficient	*r_s_*	*r_s_^2^* (%)	
Physical function measures							
Grip strength (kg)	−0.151	0.127	1.61	−0.276	−0.147	2.16	3.77
Log transformed force plate postural sway 95% area (in.^2^) - Firm	−0.072	−0.233	5.41	0.926	0.227	5.17	10.58
Log transformed force plate postural sway 95% Area (in.^2^) - Foam	0.306	−0.239	5.72	−1.051	−0.454	20.63	26.36
eSPPB	0.896	0.948	89.82	−0.376	0.074	0.54	90.36
Log transformed 400-m walk pace (m/s)	0.126	0.657	43.17	−0.221	−0.098	0.96	44.13
Dual task pace (m/s)	0.207	0.694	48.14	0.722	0.384	14.78	62.92
Canonical correlation (*R*_c_)			0.51			0.44	
Squared canonical correlation [*R*_c_^2^(%)]			26.31			19.52	
Cognitive measures							
MoCA Adjusted Score	−0.048	0.091	0.82	0.205	0.381	14.55	15.37
Semantic fluency animals (no. in 60 s)	0.531	0.497	24.72	−0.318	−0.071	0.50	25.22
Semantic fluency vegetables (no. in 60 s)	−0.080	0.203	4.12	−0.199	−0.080	0.64	4.76
Verbal fluency (L words) (no. in 60 s)	0.045	0.105	1.11	0.720	0.536	28.73	29.84
Verbal fluency (F words) (no. in 60 s)	−0.281	−0.149	2.23	−0.318	0.164	2.69	4.93
Craft immediate recall (no.)	−0.546	−0.209	4.37	0.426	0.547	29.92	34.30
Craft delayed recall (no.)	0.347	−0.102	1.05	0.072	0.458	21.01	22.05
DSC (no. in 90 s)	0.493	0.625	39.09	0.142	0.318	10.09	49.19
AVLT short delay recall, Trial 6 (no.)	0.121	−0.044	0.19	−0.587	0.162	2.63	2.82
AVLT delayed recall (no.)	−0.322	−0.151	2.29	0.627	0.322	10.34	12.64
Log transformed TMT A (sec)	0.423	−0.187	3.50	−0.190	−0.296	8.78	12.28
Log transformed TMT B (sec)	−0.527	−0.571	32.63	−0.160	−0.456	20.80	53.43
Flanker (log of ratio of medians (sec))	−0.217	−0.265	7.02	−0.025	−0.107	1.14	8.16

Underlined effects have *r*_s_^2^ > ~15%; Hierarchical Tests of canonical correlations (1–6: *p* = 0.0001; 2–6: *p* = 0.0211; 3–6: *p* = 0.2545; 4–6: *p* = 0.6252; 5–6: *p* = 0.9125; 6: *p* = 0.9832). The standardized function coefficients (Std. Coef.) define the linear combinations used to construct each synthetic variable. The structure coefficients (*r*_s_) measure the bivariate correlation between an observed individual variable and the synthetic variable that incorporates that measure. The square of the structure coefficients × 100 (*r*_x_^2^) measures the proportion of variance shared by the observed variable and the created synthetic variable. The communality coefficient × 100 (*h*^2^) measures the proportion of variance that each observed measure shares with the solution across the selected functions, is equal to the sum of the structure coefficients, and is informative as to the importance of the individual variable across the selected functions. The canonical correlation (*R*_c_) is the Pearson correlation between the linear functions for the physical and cognitive measures. The square of this value represents the proportion of variance shared between the cognition and physical function variables, after accounting for all previous pairs of synthetic variables.

Focusing on the cognitive variable set in Function 1, we found that semantic fluency (animals), DSC total score, and log transformed TMT B cognitive measures were the primary measures that contributed to the linear synthetic variable. Because the DSC and animals scores were inversely related to TMT B, the structure coefficient for TMT B was negative.

The 2nd function explained 19.5% of the remaining shared variance after accounting for the first function. The coefficients in Table 2 for this function suggested that this function primarily relates a synthetic cognition variable with primary contributions of the MoCA adjusted score, Verbal Fluency L words, Craft story immediate and delayed recall measures, and log transformed TMT B to the log transformed foam-based force plate postural sway time and the Dual Task. We labeled this function as “complex motor tasks and complex cognitive tasks” many of which provided an additional challenge to executive function and cognitive attention. Because software performing valid missing data imputation and analysis methods appropriate for CCA are very limited, we performed a sensitivity analysis to increase our sample size. This was done by dropping the grip strength and flanker variables to gain data from 4 participants, bringing the analytical sample with complete data to 93% of the baseline sample (*n* = 176). There were no substantial changes to the results.

## Discussion

There is a growing body of literature suggesting a relationship between gait speed and cognition, and moreover that dysfunction in both domains may predict onset of dementia (Inzitari et al., 2007; Clouston et al., 2013; Chou et al., 2019; Jayakody et al., 2019, 2021). However, which types of cognitive measures are associated with gait speed has varied across studies (Morris et al., 2016) and there are more limited analyses relating other measures of physical performance to specific tests of cognitive performance. This study is the first to apply CCA to 18 tests of cognitive and physical performance to determine whether there are any important underlying synthetic functions relating these assessments in cognitively healthy older adults. The first function explained a large proportion (26.3%) of the joint variability between these sets of variables and included tests of cognitive and physical speed. The second function explained 19.5% of the remaining variance and included complex motor tasks and challenges to cognitive function including executive function and cognitive attention. The relative grouping of these measures may suggest the involvement of shared underlying neurophysiologic pathways required to accomplish those tasks.

Slowed gait speed has been previously shown to predict decline in processing speed as measured by the Symbol Search and DSC (Inzitari et al., 2007; Chou et al., 2019; Jayakody et al., 2019). Similarly, in our study, multiple timed assessments that included gait speed were associated with several timed assessments of cognition related to processing speed in Function 1. The eSPPB included both usual 4-m gait speed and narrow walking pace, and the eSPPB score was highly correlated with the 400-m walking pace. The Dual Task also measured walking pace on the GaitRITE mat while completing a cognitive verbal task. All three of the correlated cognitive tasks were also timed assessments that partially capture processing speed: semantic fluency (naming animals), DSC, and TMT B. Participants in BNET were cognitively intact at baseline, so the underlying association observed between these measures of gait speed and cognitive speed may be intrinsic rather than due to impairment in cognition and could suggest a common shared neurophysiologic pathway related to speed. Moreover, injury to such a pathway could result in dual impairments in gait speed and cognitive processing. Concurrent declines in gait speed and cognition could place those individuals at significantly greater risk of incident dementia compared to those who decline in gait speed or cognitive function alone (Collyer et al., 2022). Study of the neuropathophysiology underlying these associations will be an important next step toward identification of potential upstream targets that could be intervened upon to prevent cognitive disability.

As one ages, gait also increasingly relies on higher order executive function, which may explain why performing a cognitive verbal dual task while walking was not only associated with processing speed (Function 1) but also executive function and cognitive attention (Function 2; Ezzati et al., 2015). For example, dual task was related to cognitive tasks like semantic fluency and verbal fluency that draw on both speed and executive function. A recent study by Holtzer and colleagues used principal components analysis to determine cognitive factors, and then used multiple regression analyses to examine the relationship between the cognition factors and gait velocity with and without interference by dual task in a cohort of cognitively normal older adults (Holtzer et al., 2006). Most notably, they found that speed/executive attention and memory both predicted gait velocity not only under usual conditions but also whenever there was interference by introduction of a secondary verbal task (i.e., dual task). A recent meta-analysis also found strong evidence that mild cognitive impairment was associated with impaired gait in particular during dual task conditions (Bahureksa et al., 2017), which may suggest a stronger association between early cognitive dysfunction and gait dysfunction under conditions that make competing demands on attention or that challenge both physical and cognitive reserve. Older adults with slower gait speed, particularly during dual task, are also at particularly higher risk of incident falls (Verghese et al., 2002). Similarly, cognitive impairment may increase one’s risk for falls but which specific domains are responsible for falls is not known (Shaw, 2002; Allali et al., 2017). The relationship between cognition, gait speed, and falls is complex and multifactorial. Future studies should consider if concurrent decrements in dual task gait speed and cognitive tests that challenge speed and executive function may help to identify a subgroup of older adults at substantially greater risk of not only cognitive impairment but also mobility disability, including falls.

The predominant complex motor task in the second function was maintaining postural stability on the foam-based force plate. Similar to the dual task, which provided an additional cognitive challenge while walking, standing on the foam rather than firm surface provided an additional stress to cognitive attention which is otherwise known to decline with age (Craik and Byrd, 1982). This may explain why postural sway on the foam surface was associated with cognitive assessments of executive function such as verbal fluency and log transformed TMT B. Older adults have been shown to have significantly worse postural control on compliant, unstable surfaces (e.g., foam) relative to younger adults (Hsiao et al., 2020). Moreover, in one study, the association between poor executive function and falls was mediated by postural sway (Taylor et al., 2017), which may correspond to the connection between postural sway and executive function observed in Function 2 in our study. More global cognitive (e.g., MOCA) and memory tasks were also associated, possibly due to the complex nature of these assessments, but further research is needed to elucidate what neurophysiologic pathways may relate these measures.

Although the underlying neuropathophysiology connecting the physical and cognitive measures in Functions 1 or 2 cannot be directly ascertained by CCA, the groupings of particular measures with gait speed or complex motor tasks like postural sway on a foam surface may suggest future directions for further exploration on neuroimaging. A growing body of literature has examined whether associations between mobility, especially gait speed, and brain structure are explained by cognitive measures (Wilson et al., 2019). In some cases, adjusting for cognition attenuated the relationship between gait velocity and specific regions of the brain (Wilson et al., 2019) or other structural imaging measures such as beta amyloid burden (Nadkarni et al., 2017). For example, the relationship between gait and hippocampal volume was attenuated after adjustment for verbal memory (Ezzati et al., 2015). Similarly, after adjustment for MMSE there was no association of gait speed with frontal and parietal lobe gray matter volume, but there was a persistent relationship with sensorimotor cortex, insula, thalamus, basal ganglia, and caudate nucleus volumes (Dumurgier et al., 2012). Additional adjustment for TMT-A also had little impact on the association of gait speed with subcortical volumes of the caudate nucleus and basal ganglia. In the Health, Aging and Body Composition study, larger cognitive cerebellar gray matter volume were associated with faster gait speed but this was not independent of DSC scores, and larger sensorimotor cerebellar volume was also associated with higher DSC but not gait (Nadkarni et al., 2014). Meanwhile vestibular volumes were associated with neither gait nor DSC. The authors concluded that information processing speed may influence the association between gait speed and cerebellar gray matter volumes, especially in the cognitive sub-region. These findings track well with our finding of a correlation between cognitive measures of processing speed and gait speed in Function 1.

Similarly, older adults are known to experience decrements in postural and volitional balance control (Kanekar and Aruin, 2014) that are more pronounced in those with mild cognitive impairment (Bahureksa et al., 2017). Reduced gray matter volumes in the brainstem and cerebellum have been significantly associated with reduced postural control (Kannan et al., 2022). Compared to cognitively normal older adults, those with mild cognitive impairment and Alzheimer’s dementia have also been shown to have more vestibular impairment, which in turn was associated with lower hippocampal volumes (Cohen et al., 2022). One limitation of the current literature is that most of the imaging methodologies applied in the context of mobility and cognition have examined structural rather than functional metrics. Exploration of particular pathways *via* network science, however, may provide better insight into how and why specific aspects of gait speed, balance, and cognition are functionally related, and will be the focus of future analyses.

## Limitations

A notable limitation of this study was the exclusion of participants who had evidence of substantial cognitive impairment (i.e., MOCA scores 21–25 who were deemed ineligible by a neuropsychologist or MOCA scores of 20 or lower). Similarly, potential participants were excluded if they had substantial mobility restrictions due to prior amputations or joint replacement, or if they depended on a walker or another person to ambulate. Since all included participants had higher cognitive and physical function at baseline, this likely restricted our ability to detect correlations between very poor cognitive and physical performance on these tests. However, it is possible that older adults with substantial physical or cognitive impairment would not have been able to complete these complex motor and cognitive tasks. Moreover, our findings highlight that there is correlation between these cognitive and physical measures even when assessed in a cohort of highly functional older adults. Thus, these early markers of dysfunction may be preclinical and hence upstream of disability, suggesting a possible point of intervention for future work. The reasons that particular tests grouped into Function 1 versus 2 are likely multifactorial and may not be fully explained by the more general names we ascribed to these functions. While some common themes were noted, such as the importance of speed to the tests of Function 1, there were also timed cognitive assessments that did not group into Function 1. Similarly, executive function was not exclusively important to the tests in Function 2 and why certain assessments were not highly correlated with one of the functions is not well understood but should be further investigated. Whether common neurologic pathways may underlie these groups of functions will be a focus of future study.

## Conclusion

In summary, we applied CCA to identify two connected groups of cognitive and physical function tasks in a cross-sectional cohort of cognitively intact, healthy older adults. The predominant function included speed related tasks in gait and cognition, while the second function included complex motor and cognitive tests. Future studies should investigate whether common underlying neurologic pathways are shared by these functions and may provide a point of intervention to prevent downstream disability.

## Data availability statement

The raw data supporting the conclusions of this article will be made available by the authors, without undue reservation.

## Ethics statement

The studies involving human participants were reviewed and approved by Wake Forest Baptist Health Institutional Review Board. The patients/participants provided their written informed consent to participate in this study.

## Author contributions

SK, MM, and EH: conceptualization. MM, EH, HC, and AT: methodology. MM, SK, CH, PL, HC, and AT: validation. MM, EH, AT, PL, SK, and CH: investigation. AT, MM, and EH: writing. AT, MM, EH, HC, CH, PL, and SK: editing manuscript. SK and PL: funding acquisition. All authors have read and agreed to the published version of the manuscript.

## Funding

This research was supported by the National Institutes of Health (R01AG052419 and 3R01AG052419-02S1), the Wake Forest Claude D. Pepper Center (P30AG021332), and the Wake Forest Clinical and Translational Science Awards (UL1TR001420). AT receives support from the National Eye Institute (K23EY030897).

## Conflict of interest

AT is a consultant for Topcon Medical Inc. which had no relationship to this study.

The remaining authors declare that the research was conducted in the absence of any commercial or financial relationships that could be construed as a potential conflict of interest.

## Publisher’s note

All claims expressed in this article are solely those of the authors and do not necessarily represent those of their affiliated organizations, or those of the publisher, the editors and the reviewers. Any product that may be evaluated in this article, or claim that may be made by its manufacturer, is not guaranteed or endorsed by the publisher.
