# Corrigendum: Examining the intersection of cognitive and physical function measures: Results from the brain networks and mobility (B-NET) study

**DOI:** 10.3389/fnagi.2023.1166863

**Published:** 2023-03-01

**Authors:** Atalie C. Thompson, Michael E. Miller, Elizabeth P. Handing, Haiying Chen, Christina E. Hugenschmidt, Paul J. Laurienti, Stephen B. Kritchevsky

**Affiliations:** ^1^Department of Surgical Ophthalmology, Wake Forest School of Medicine, Winston-Salem, NC, United States; ^2^Section on Gerontology and Geriatric Medicine, Department of Internal Medicine, Sticht Center for Healthy Aging and Alzheimer's Prevention, Wake Forest School of Medicine, Winston-Salem, NC, United States; ^3^Division of Public Health Sciences, Wake Forest School of Medicine, Winston-Salem, NC, United States; ^4^Department of Human Development and Family Studies, Colorado State University, Fort Collins, CO, United States; ^5^Department of Biostatistics and Data Sciences, Wake Forest School of Medicine, Winston-Salem, NC, United States; ^6^Department of Radiology, Wake Forest School of Medicine, Winston-Salem, NC, United States

**Keywords:** cognitive function, mobility, aging, canonical correlation analysis, physical function

In the published article, there was an error in Table 1 as published. Two participants were miscategorized as American Indian or Alaskan Native and should have been categorized as Caucasian or White race with Hispanic ethnicity. In addition, the standard deviation for age was incorrectly written as 4.74 and should have been 4.72. The corrected Table 1 appears below and includes updated race/ethnicity variable labels.

**Table 1:** Descriptive statistics from participants at baseline in the BNET study.

	**Overall (*N* = 192) Mean (SD); range**
Age	76.43 (4.72); 70 to 90
**Sex**	
Women	108 (56.2)
Men	84 (43.8)
**Race/Ethnicity**	
Caucasian or White/Non-Hispanic	171 (89.1)
African American or Black/Non-Hispanic	18 (9.4)
Caucasian or White/Hispanic	2 (1.0)
Asian/Non-Hispanic	1 (0.5)
BMI	28.39 (5.63); 15.7 to 59.8
Years of education	15.68 (2.45); 12 to 25
**Cognitive measures**	
MoCA adjusted score	25.64 (2.20); 21 to 30
Semantic fluency: Animals (no. in 60 s)	18.78 (4.82); 7 to 34
Semantic fluency: Vegetables (no. in 60 s)	13.26 (3.87); 0 to 26
Verbal fluency: F words (no. in 60 s)	12.33 (3.94); 3 to 26
Verbal fluency: L words (no. in 60 s)	13.23 (4.02); 4 to 28
CRAFT immediate recall (no.)	21.03 (5.99); 7 to 35
CRAFT delayed recall (no.)	18.67 (5.74); 7 to 34
DSC (no. in 90 s)	55.18 (12.20); 21 to 87
AVLT short delay recall, Trial 6 (no.)	8.37 (3.20); 0 to 15
AVLT delayed recall (no.)	7.94 (3.46); 0 to 15
TMT A (sec)	36.75 (11.15); 18 to 89
TMT B (sec) (*N* = 191)	98.70 (43.96); 36 to 300
Flanker (log of ratio of medians) (*N* = 189)	0.11 (0.08); −0.03 to 0.39
**Physical function measures**	
Maximum grip strength (kg) (*N* = 189)	28.80 (9.78); 8 to 52
Force plate postural sway 95% Area (in.^2^) – Firm (*N* = 188)	0.37 (0.34); 0.07 to 2.40
Force plate postural sway 95% Area (in.^2^) – Foam (*N* = 188)	1.18 (0.82); 0.34 to 8.68
eSPPB score (*N* = 190)	2.00 (0.52); 0.48 to 3.26
400 m walk pace (m/s)	1.27 (0.43); 0.31 to 4.17
Dual Task pace (m/s) (*N* = 186)	1.07 (0.21); 0.55 to 1.67

MoCA, Montreal cognitive assessment; DSC, Digit symbol coding; AVLT, Auditory verbal learning test; TMT, Trail making test.

The authors apologize for this error and state that this does not change the scientific conclusions of the article in any way. The original article has been updated.

