# Supplementary material for: Examining the intersection of cognitive and physical function measures: Results from the brain networks and mobility (B-NET) study
Source: Front Aging Neurosci. 2023 Feb 2;15:1090641. doi: 10.3389/fnagi.2023.1090641 (PMC9932333; doi:10.3389/fnagi.2023.1090641)
Supplement: Supplementary file 1 [file Table_1.doc]

**Supplemental Table 1: Pearson Correlations Adjusted for Sex and Education**

|  | **MOCA** | **Category Frequency Animals** | **Category Frequency Vegetables** | **Verbal Fluency**  **(L Words)** | **Verbal Fluency**  **(F Words)** | **Craft Immediate Recall** | **Craft Delayed Recall** | **DSC** | **AVLT Short Recall; Trial 6** | **AVLT Delayed Recall** | **TMT A^a^** | **TMT B^a^** | **Flanker^b^** | **Grip Strength** | **Force Plate (Firm)^a^** | **Force Plate (Foam)^a^** | **eSPPB** | **400-m walk^a^ (m/sec)** |
| --- | --- | --- | --- | --- | --- | --- | --- | --- | --- | --- | --- | --- | --- | --- | --- | --- | --- | --- |
| **Category Frequency Animals** | 0.18 | 1.00 |  |  |  |  |  |  |  |  |  |  |  |  |  |  |  |  |
| **Category Frequency Vegetables** | 0.23 | 0.43 | 1.00 |  |  |  |  |  |  |  |  |  |  |  |  |  |  |  |
| **Verbal Fluency**  **(L Words)** | 0.25 | 0.25 | 0.13 | 1.00 |  |  |  |  |  |  |  |  |  |  |  |  |  |  |
| **Verbal Fluency**  **(F Words)** | 0.23 | 0.14 | 0.06 | 0.60 | 1.00 |  |  |  |  |  |  |  |  |  |  |  |  |  |
| **Craft Immediate Recall** | 0.17 | 0.08 | 0.07 | 0.03 | 0.03 | 1.00 |  |  |  |  |  |  |  |  |  |  |  |  |
| **Craft Delayed Recall** | 0.15 | 0.11 | 0.10 | 0.03 | 0.08 | 0.86 | 1.00 |  |  |  |  |  |  |  |  |  |  |  |
| **DSC** | 0.19 | 0.25 | 0.12 | 0.14 | 0.07 | 0.02 | 0.03 | 1.00 |  |  |  |  |  |  |  |  |  |  |
| **AVLT Trial 6** | 0.29 | 0.15 | 0.19 | 0.09 | 0.04 | 0.28 | 0.35 | 0.11 | 1.00 |  |  |  |  |  |  |  |  |  |
| **AVLT Delayed Recall** | 0.29 | 0.15 | 0.18 | 0.03 | 0.06 | 0.30 | 0.34 | 0.09 | 0.84 | 1.00 |  |  |  |  |  |  |  |  |
| **TMT A^a^** | -0.05 | -0.31 | -0.23 | -0.16 | -0.04 | 0.00 | -0.05 | -0.48 | -0.11 | -0.10 | 1.00 |  |  |  |  |  |  |  |
| **TMT B^a^** | -0.24 | -0.15 | -0.19 | -0.16 | -0.04 | -0.15 | -0.15 | -0.43 | -0.14 | -0.15 | 0.41 | 1.00 |  |  |  |  |  |  |
| **Flanker^b^** | -0.06 | 0.01 | -0.22 | -0.18 | -0.04 | 0.04 | 0.03 | -0.08 | -0.05 | 0.00 | 0.08 | 0.10 | 1.00 |  |  |  |  |  |
| **Grip Strength** | 0.10 | 0.09 | 0.08 | 0.00 | 0.10 | -0.03 | -0.05 | 0.02 | 0.04 | 0.01 | -0.03 | -0.07 | -0.09 | 1.00 |  |  |  |  |
| **Force Plate (Firm)^a^** | -0.03 | 0.06 | -0.09 | 0.03 | -0.04 | 0.15 | 0.14 | -0.10 | -0.04 | 0.00 | -0.03 | 0.08 | 0.14 | -0.08 | 1.00 |  |  |  |
| **Force Plate (Foam)^a^** | -0.17 | 0.06 | -0.09 | -0.15 | -0.13 | -0.08 | -0.08 | -0.16 | -0.10 | -0.07 | 0.02 | 0.24 | 0.14 | -0.10 | 0.65 | 1.00 |  |  |
| **eSPPB** | 0.10 | 0.24 | 0.12 | 0.08 | -0.03 | -0.06 | 0.00 | 0.31 | 0.01 | -0.05 | -0.07 | -0.32 | -0.13 | 0.22 | -0.31 | -0.46 | 1.00 |  |
| **400-m walk (m/sec)^a^** | 0.01 | 0.09 | 0.13 | 0.07 | 0.00 | -0.12 | -0.08 | 0.21 | -0.03 | -0.12 | -0.05 | -0.21 | -0.16 | 0.31 | -0.25 | -0.24 | 0.58 | 1.00 |
| **Dual Task (m/sec)** | 0.11 | 0.15 | 0.07 | 0.12 | -0.01 | -0.05 | -0.07 | 0.32 | 0.02 | 0.03 | -0.18 | -0.30 | -0.20 | 0.34 | -0.29 | -0.36 | 0.62 | 0.56 |

Blue shading indicates correlation matrix between cognitive measures. Pink shading indicates correlation matrix between measures of physical function. No shading indicates correlation matrix between cognitive and physical function measures.

^a^Log transformed; ^b^log of ratio of medians
